# Antibacterial Properties of Methanolic Leaf Extracts of *Melia azedarach* L. against Gram-Positive and Gram-Negative Pathogenic Bacteria

**DOI:** 10.3390/microorganisms11082062

**Published:** 2023-08-11

**Authors:** Soraya Naila Touzout, Abderrahmen Merghni, Aicha Laouani, Halima Boukhibar, Rawaf Alenazy, Abdulmohsen Alobaid, Mustafa Alenazy, Mossadok Ben-Attia, Khaled Saguem, Safia El-Bok

**Affiliations:** 1 Laboratory of Biodiversity, Biotechnologies and Climate Change (LR11/ES09), Faculty of Sciences of Tunis, University of Tunis El-Manar, Tunis 2092, Tunisia; sorayatouzout@gmail.com (S.N.T.); boukhibar.saida@gmail.com (H.B.); safia.elbok@fst.utm.tn (S.E.-B.); 2 Laboratory of Antimicrobial Resistance LR99ES09, Faculty of Medicine of Tunis, University of Tunis El Manar, Tunis 1007, Tunisia; 3 Laboratory of Metabolic Biophysics and Applied Pharmacology, Faculty of Medicine of Sousse, University of Sousse, Sousse 4002, Tunisia; aicha.laouani@famso.u-sousse.tn (A.L.); khaledsaguem@famso.u-sousse.tn (K.S.); 4 USCR Analytical Platform UHPLC-MS &Research in Medicine and Biology, Faculty of Medicine of Sousse, University of Sousse, Sousse 4002, Tunisia; 5 Department of Medical Laboratory, College of Applied Medical Sciences-Shaqra, Shaqra University, Shaqra 11961, Saudi Arabia; 6 Department of Medical Laboratory, Aliman General Hospital-Riyadh, Ministry of Health, Riyadh 12684, Saudi Arabia; absaalobaid@moh.gov.sa; 7 Al-DAWAA Medical Company, Riyadh 14814, Saudi Arabia; mustafa05616@gmail.com; 8 Environment Biomonitoring Laboratory (LR01/ES14), Faculty of Sciences of Bizerte, University of Carthage, Zarzouna 7021, Tunisia; mossadok.benattia@fsb.ucar.tn

**Keywords:** *M. azedarach* L., methanolic extract, HPLC-DAD, antibacterial potential, mode of action

## Abstract

*Melia azedarach* L., a *Meliaceae* family tree, is widely used in traditional folkloric medicine for its pharmaceutical properties. In the present study, we investigated the phytochemical composition of four methanolic leaf extracts of *M. azedarach* of various origins (Algeria and Tunisia) using high-performance liquid chromatography (HPLC). The antibacterial efficacy and mechanisms of action against Gram-positive and Gram-negative pathogenic microorganisms were then evaluated. Our findings revealed a presence of phenolic acids and flavonoids, such as gallic acid, chlorogenic acid, caffeic acid, hyperoside, isoquercetin, quercetin, and isorhamnetin both in Algerian and Tunisian localities, with an abundance of phenolic acids compared to flavonoids. Additionally, the studied extracts exhibit a broad spectrum of antibacterial activities, with MIC values ranging from 31.25 mg/mL to 125 mg/mL. Methanolic leaf extracts of *M. azedarach* from Algeria exhibited more potent biofilm eradication, with a percentage of inhibition reaching 72.17% against the *S. aureus* strain. Furthermore, inhibitory concentrations of tested substances, particularly the extract from the Relizane area, were capable of disrupting the membrane integrity of the treated bacteria as well as producing oxidative stress through ROS generation. Likewise, our results reveal that plant extract induces lipid peroxidation by raising MDA levels in comparison to untreated cells, particularly with the plant extract of Blida. *M. azedarach* extracts also reduced the synthesis of antioxidant enzymes (CAT and SOD). Our findings illustrate that *M. azedarach* remains a plant with significant antibacterial potential and distinct mechanisms of action that are closely related to the origins of this specimen.

## 1. Introduction

The emergence of bacterial resistance poses a serious global danger. Several infectious diseases caused by resistant bacteria induce higher morbidity, mortality, and treatment costs [1]. Despite the fact that antimicrobials are required to treat a variety of infection-related disorders, these medications are well known for their negative side effects, such as allergic reactions and alteration of the normal microbiota of patients [2]. Furthermore, it has been shown that synthetic antibiotics are only effective in treating a third of the known infectious diseases [3]. In light of these growing therapeutic challenges, it has become urgently necessary to find new compounds as prototypes for the development of less toxic and more potent medications to combat infectious diseases by limiting the proliferation of microbes [4,5].

Natural products derived from medicinal plants have been shown to contain significant numbers of bioactive molecules with potent antibacterial activities [6,7]. *Melia azedarach*, a Meliaceae tree, is widely recognized for its several medicinal properties, including antiviral, antimalarial, antifungal, and antibacterial activities [8]. Whereas all parts of this tree have medicinal potential, the leaves have the most pharmaceutical advantages [6]. *M. azedarach* was traditionally used in Ayurvedic medicine for its bactericidal activity, and may therefore provide a new alternative antimicrobial treatment [9]. Furthermore, this plant contains abundant concentrations of phenolic compounds [10]. Interestingly, polyphenolic flavonoids, which are prevalent in herbals [7,11] and medicinal plants, have a variety of uses in the pharmaceutical sector, particularly for their effectiveness against Gram-positive and Gram-negative bacteria [12,13].

Several alternative antimicrobials that target oxidative stress in pathogenic bacteria have recently been researched and have proven to be promising [5]. Thus, bacterial oxidative stress is caused by the accumulation of reactive oxygen species (ROS) [14], which affect several targets in exposed cells at the same time, including proteins, lipids, and nucleic acids [15]. Consequently, these cell damages cause ROS-mediated toxicity and lead to bacterial death [16].

The current study aims to investigate the phytochemical composition of four methanolic leaf extracts of *M. azedarach* from different geographic localities (Algeria and Tunisia) using high-performance liquid chromatography (HPLC), as well as to evaluate their antibacterial and antibiofilm activity and to assess their effects on bacterial membrane integrity, reactive oxygen species generation, and lipid peroxidation. The impact of the tested extracts on oxidative stress enzyme (superoxide dismutase and catalase) production was also investigated.

## 2. Materials and Methods

### 2.1. Plant Origins

*M. azedarach* L. leaves were sampled in Algeria, from two localities: Blida (36°48″ N, 2°83″ E) and Relizane (35°44″ N, 0°33″ E). Additionally, this specimen was collected from two Tunisian regions: Bizerte (37°16′ N, 9°52′ E) and Sousse (35°49′ N, 10°36′ E). Botanical identification of the plant was carried out at the Faculty of Science and Technology, Tunis El Manar University (UTM).

### 2.2. Preparation of the Methanolic Extracts

The leaves of *M. azedarach* from the different geographic zones were air-dried at room temperature for 48 h and then mechanically ground into a fine powder. The obtained powders were used for preparation of the methanolic extract (80% *v*/*v*). To remove the solvent from the final extracts, a rotary evaporator (Buchi Rotavapor R-215, BUCHI, Shanghai, China) was used prior to chemical analysis and bioassays.

### 2.3. HPLC-DAD Analyses

The chromatographic analyses of methanolic leaf extracts of *M. azedarach* sampled from different regions of Algeria (Relizane and Blida) and Tunisia (Sousse and Bizerte) were performed using an HPLC (high-Performance liquid chromatography) device of the Agilent 1200 type (Agilent, Santa Clara, CA, USA), which was controlled by a computer. The separation was carried out on an Agilent HPLC system supplied with a diode array detector (DAD). For the separation, a Kinetex Evo C18 analytical column (Agilent, Santa Clara, CA, USA) was used at a temperature of 40 °C. The injection volume was 20 µL and peaks were monitored at 254 nm. Samples were filtered through a 0.22 µm filter before injection. The mobile phase A was formed by 99% water and 1% of formic acid, and mobile phase B was formed by a combination of acetonitrile and formic acid (1%) with a flow rate of 1 mL/min. The chromatographic conditions were as follows: 90% A, 10% B (0 min); 80% A, 20% B (20 min); 75% A, 25% B (30 min); 65% A, 35% B (40 min); and 90% A, 10% B (50 min). Under these conditions, the pump generated a pressure of approximately 150 bars. The measurement of optical density ensured spectrophotometric detection of the analytes at a fixed wavelength of 254 nm. The identification of phenolic compounds was conducted by comparing their retention times and UV spectra with the available authentic standards. The quantification was estimated by comparing the area of the peak of interest with that obtained in a chromatogram of the standard corresponding to a known concentration.

### 2.4. Disc Diffusion Assay

Two Gram-positive (*Staphylococcus aureus* ATCC 25923 and *Staphylococcus epidermidis* ATCC 14990) and two Gram-negative (*Escherichia coli* ATCC 25922 and *Pseudomonas aeruginosa* ATCC 27853) bacterial strains were used to test the antibacterial activity of the various leaf extracts of *M. azedarach*. Each strain’s bacterial suspension was adjusted to have an optical density of about 0.5 McFerland. After Mueller–Hinton (MH) agar plating using sterilized swabs, a sterile Wattman paper disc 6 mm in diameter was deposited into each plate. Then, a volume of 10 µL from each plant extract was added. A standard Gentamicin^®^ disc was used as the positive control disc, and the experiment was performed in triplicate. Following the incubation of MH plates for 24 h at 37 °C, the diameter of inhibition around each disk was measured [17].

### 2.5. Determination of the Minimal Inhibitory and Bactericidal Concentrations

The minimum inhibitory concentration (MIC) of *M. azedarach* leaf extracts from different provenances against bacterial strains was assessed as previously described [18]. Stock solutions of *M. azedarach* extracts were prepared aseptically in DMSO 10% (*v*/*v*), then transferred to sterile 96-well microtiter plates containing the MH broth. Each plant extract was then subjected to a series of cascade dilutions to achieve a final concentration ranging from 0.5 to 250 mg/mL. The final volume in each microplate well was fixed to 200 µL, containing a specific concentration of each *M. azedarach* extract, the growth medium (MH), and the bacterial inoculum (0.5 McF). Following incubation for 24 h at 37 °C, 20 μL per well of MTT (methyl thiazolyl-diphenyl tetrazolium bromide, Sigma-Aldrich, Burlington, MA, USA) solution (0.5 mg/mL) was added for better visualization of the bacterial growth, then microplates were further incubated (37 °C for 3 h). Bacterial growth was assessed by observing the color change in each well from yellow to purple. The lowest concentration of the samples that visually inhibited the bacteria was considered to be MIC [19].

The minimum bactericidal concentration (MBC) value was determined by withdrawing 10 µL from each well with no detectable growth and inoculating into MH agar plates, followed by further incubation for 24 h at 37 °C. The MBC value was defined as the lowest concentration for which 99% of bacteria were eradicated [20].

### 2.6. Antibiofilm Activity

The antibiofilm activity of *M. azedarach* leaf extracts was evaluated using the crystal violet (CV) staining test. Mature biofilms, 48 from each tested bacterial strain, were established in sterile 96-well microplates as previously described [21]. Following incubation, non-adherent bacterial cells were removed and the established biofilms were treated with various concentrations (MIC, MIC × 2, and MIC × 4) of the studied extracts, which were prepared in DMSO and brain heart infusion (BHI) broth [22]. After 24 h of incubation, biofilms were stained with 100 μL of CV for 30 min, then plates were washed and air-dried and the biofilm biomass was measured using a microplate reader. The formula below calculates the biofilm eradication percentage: [(OD growth control-OD sample)/OD growth control] × 100 [23]. Untreated biofilms represent the negative controls.

### 2.7. Membrane Integrity

In order to assess the membrane integrity, bacterial cultures (0.5 McF) were treated with different extracts of *M. azedarach* for 2 h at 37 °C. After incubation, the treated bacterial cultures were pelleted by centrifugation (3000 rpm, for 10 min), washed twice, and then resuspended in 0.85% (*w*/*v*) saline [16]. Membrane leakage was finally quantified by measuring the optical density with a spectrophotometer at two different wavelengths, i.e., 260 nm and 280 nm. This test was carried out in triplicate [24].

### 2.8. Reactive Oxygen Species Production (ROS)

Using a peroxynitrite developer, 2’-7’-dichlorodihydrofluorescein diacetate (DCFH-DA) (Sigma Aldrich, Gillingham, UK), which can identify a wide range of reactive oxygen species (ROS) including nitric oxide and hydrogen peroxide, the levels of ROS generation by bacterial strains *S. aureus* and *E. coli* exposed to various extracts of *M. azedarach* was assessed [25]. The various plant extracts were added to each bacterial culture (0.5 McF), and DCFH-DA was added for a fixed final concentration of 5 M in saline solution (0.85%). Following incubation for 24 h at 37 °C, the fluorescence emission from DCFH-DA was measured at a wavelength of 525 nm using a microtiter plate reader with an excitation wavelength of 485 nm. Each experiment was performed three times [26].

### 2.9. Evaluation of Lipid Peroxidation

Malondialdehyde (MDA) is a naturally occurring by-product of polyunsaturated fatty acid lipid peroxidation induced by ROS. Therefore, it is frequently used as a sign of oxidative stress. MDA generation was measured by incubating a bacterial culture at 37 °C for 24 h with different *M. azedarach* leaf extracts. A volume of 100 µL from each treated culture was added to 100 µL of the SDS lysis solution and incubated at room temperature for 5 min after incubation. The mixtures were then exposed to a thiobarbituric acid (TBA) reagent for 60 min at 95 °C to form a compound with MDA. To stop the reaction, each combination was centrifuged at 3000 rpm for 15 min after being cooled to room temperature in an ice bath for 5 min. MDA production was measured using a microtiter plate reader at a wavelength of 532 nm. This test was performed three times [16].

### 2.10. Assessment of Antioxidant Enzyme Activity

After subjecting *S. aureus* and *E. coli* cultures to various *M. azedarach* leaf extracts for 24 h at 37 °C, the treated bacteria suspensions were centrifuged (3000 rpm for 10 min). The resultant pellet was then washed twice with PBS and the antioxidant enzyme activity was assessed [27].

The superoxide dismutase (SOD) activity was evaluated based on the ability of this enzyme to inhibit the anti-oxidation of pyrogallol at 420 nm. A volume of 0.1 mL of each bacterial extract was incubated with 2.85 mL of Tris HCl and 25 µL of pyrogallol for 30 s [28]. Then, the activity of the SOD was measured at 420 nm as follows: % inhibition = (blank Abs − Abs test)/Abs test.

For catalase (CAT) activity, bacterial extracts were added to a quartz cuvette containing 780 µL of catalase buffer (pH = 7) and 200 µL of hydrogen peroxide (20 mM). After one minute of exposure in the dark, the optical density was evaluated at 240 nm at t = 0 s and t = 1 min. One unit (U) of enzyme activity was the amount of enzyme required to degrade 1 mol of H_2_O_2_ within one second [29,30].

### 2.11. Statistical Analyses

All the experiments were performed in triplicate and the obtained data were presented as means ± standard deviations. Data were further analyzed using the SPSS 20.0 statistical package for Windows. One-way analysis of variance (ANOVA) followed by Tukey’s test was carried out to calculate the significance of the results. *p* values less than 0.05 were considered significantly statistically different.

## 3. Results

### 3.1. HPLC-DAD Analysis for Chemical Compound Identification and Quantification

**Figure 1:**
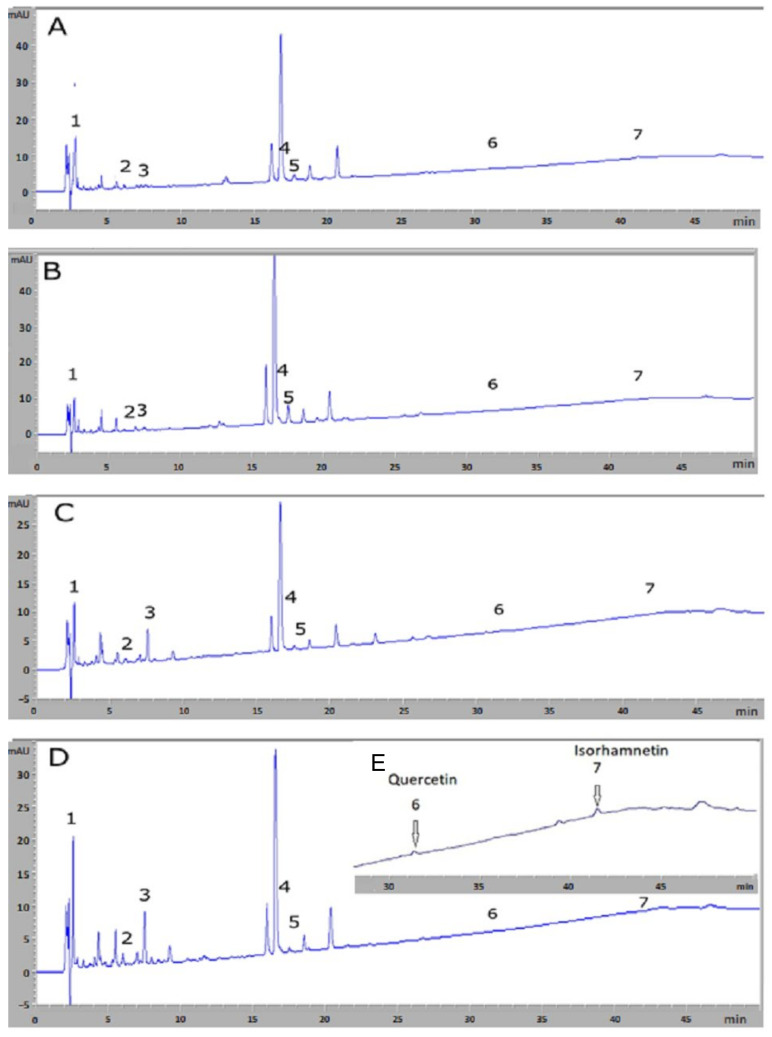
HPLC−DAD chromatographic profiles of methanolic leaf extracts of *M. azedarach* leaves from different Tunisian (Bizerte (**A**); Sousse (**B**)) and Algerian (Relizane (**C**); Blida (**D**)) localities. Panel (**E**) (upper part of (**D**)) corresponds to a widening of the small chromatographic peaks by increasing sensitivity for retention times ranging from 30 to 50 min.

The identification of chemical compounds from *M. azedarach* leaf extracts, using HPLC-DAD, are presented in Table 1. Based on HPLC analysis, the chromatographic profiles of various extracts acquired at 254 nm are shown in Figure 1. Peak identifications were confirmed by retention times (Rt), as deduced from the standard compounds listed in Table 1.

According to Table 1, seven bioactive compounds were identified in the plant extracts from the studied regions (Bizerte, Sousse, Relizane, and Blida). A qualitative similarity was observed for the seven identified compounds across the four regions, and the compounds were labeled according to their elution order. The analysis using HPLC-DAD indicated the existence of phenolic acids and flavonoids in the leaves of *M. azedarach*. The detected phenolic acids included gallic acid (Peak 1), chlorogenic acid (Peak 2), and caffeic acid (Peak 3). Hyperoside (Peak 4), isoquercetin (Peak 5), quercetin (Peak 6), and isorhamnetin (Peak 7) were flavonoids which were identified as well.

In terms of quantity and compared to the compounds which were identified, Algerian plants showed an abundance of gallic acid in the methanolic leaf extracts from the region of Blida (7601.04 ± 45.51 µg/100 mL), while quercetin, the weakest compound, was detected in trace amounts. In addition, our results showed that methanolic leaf extracts of *M. azedarach* from Algerian regions Blida and Relizane are a significant source of chlorogenic acid and of caffeic acid. Quercetin and isorhamnetin were present in smaller quantities in the studied plant.

### 3.2. Antimicrobial Activity

The results of the antibacterial activity of methanolic extracts from various provenances, firstly assessed using the disk diffusion method, are shown in Table 2.

Our findings showed varied ranges of antibacterial activity depending on the origin of the plant extracts, their concentrations, and the tested bacteria. The most important effect was recorded against Gram-positive bacteria, with inhibition zones reaching 15.5 ± 0.7 and 19 ± 0.03 mm. However, antibacterial activity was found to be less pronounced for Gram-negative bacteria (*E. coli* and *P. aeruginosa*). The effectiveness of Algerian extracts was higher than that of Tunisian ones.

Regarding the results of MICs and MBCs, most of the Tunisian *M. azedarach* extracts exhibited a bactericidal effect (MBC/MIC ≤ 4) against the tested strains (Table 3), except for Bizerte extract, which had a bacteriostatic effect on the *E. coli* strain. However, Algerian extracts showed greater bacteriostatic effects (MBC/MIC > 4) against the majority of strains.

### 3.3. Anti-Biofilm Activity

The results of the biofilm eradication potential of *M. azedarach* leaf extracts using different concentrations (MIC, MIC × 2, MIC × 4) was shown in Table 4. The tested extracts displayed different effects on the growth and development of the preformed biofilms. The highest anti-biofilm activity was obtained with an extract from Relizane, which induced inhibition of biofilm formation against *S. aureus* and *E. coli* of up to 72.17% and 60.26%, respectively. The extract from Sousse was found to be able to eradicate preformed biofilms of *S. epidermidis* and *P. aeruginosa* with percentages reaching 57.4% and 59.95%, respectively. The obtained effect was found to be concentration-dependent, with the greatest rates of biofilm reduction observed at the highest concentrations used (MIC × 4).

### 3.4. Membrane Integrity

**Figure 2:**
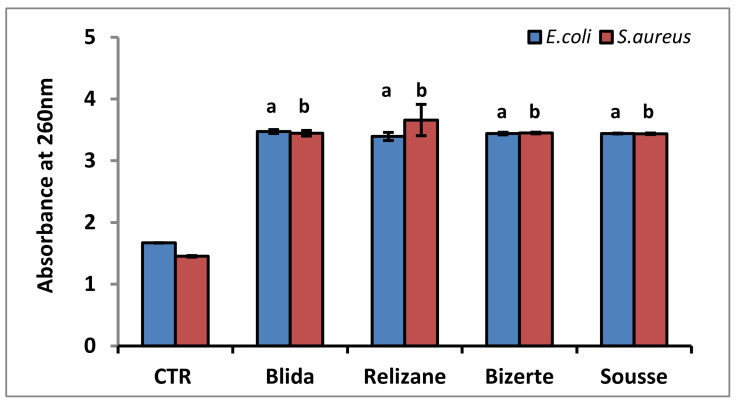

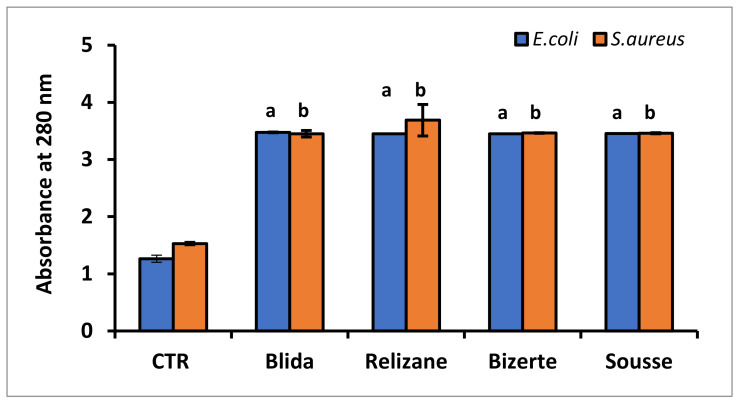
Absorbance at 260 nm and 280 nm of *E. coli* and *S. aureus* bacterial suspensions treated with leaf extracts of *M. azedarach* from various geographic zones. CTR: control. Absorbance values are expressed as mean ± SD. Comparisons of absorbances between CTR and *E. coli* or *S. aureus* treated with leaf extracts from each studied region were performed using the unpaired two-tailed Student’s *t*-test. Significant differences among the tested groups are indicated by different letters. ^a^ *p* < 0.001: *E. coli* values are significantly different from CTR; ^b^ *p* < 0.001: *S. aureus* values are significantly different from CTR.

The results regarding the integrity of the bacterial cell membranes of *S. aureus* and *E. coli* after treatment with different extracts of *M. azedarach* are presented in Figure 2.

Our results revealed a significant increase in the absorbance of the cultures of *S. aureus* and *E. coli* which were treated with the various plant extracts when compared to the control (untreated bacteria). The methanolic leaf extracts showed a 1.19- to 1.21-fold increase in optical density at 260 nm and a 1.34- to 1.35-fold increase in optical density at 280 nm compared to the *E. coli* strain control.

These extracts also showed a 1.4- to 1.49-fold increase in optical density at 260 nm and a 1.36- to 1.45-fold increase in optical density at 280 nm compared to the *S. aureus* strain controls. This increase in absorbance is due to the release of nucleic acids (260 nm) and proteins (280 nm) by the bacterial cultures compared to the controls.

### 3.5. Reactive Oxygen Species (ROS)

**Figure 3:**
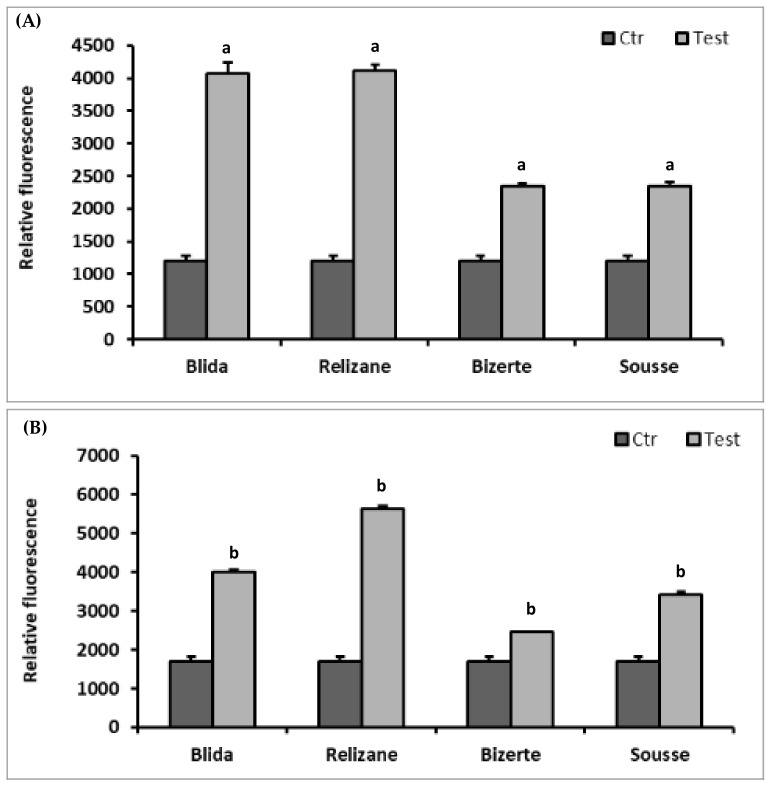
We used a DCFA-DA probe to measure the amount of intracellular ROS produced by *E. coli* (**A**) and *S. aureus* (**B**) following 24 h of exposure to leaf extracts of *M. azedarach*. The mean of relative fluorescence intensity ± SD was used to express the results. Comparisons of relative fluorescence between CTR and *E. coli* or *S. aureus* treated with leaf extracts from each studied region were performed using the unpaired two-tailed Student’s *t*-test. Significant differences among the tested groups are indicated by different letters. ^a^ *p* < 0.001: *E. coli* values are significantly different from CTR; ^b^ *p* < 0.001: *S. aureus* values are significantly different from CTR.

In this part of our study, we quantified the level of ROS production in *E. coli* and *S. aureus* after exposure to different extracts of *M. azedarach* (Figure 3).

For both bacterial species, the extracts from Blida and Relizane caused a significant increase in ROS generation in the treated strain compared to the control, as well as compared with extracts from the Tunisian regions. The highest induction of ROS was observed with the *E. coli* strain. The extracts from the regions of Bizerte and Sousse showed lower rates of production of reactive oxygen species than the Algerian extracts for both strains which were studied.

### 3.6. Evaluation of MDA Lipid Peroxidation

**Figure 4:**
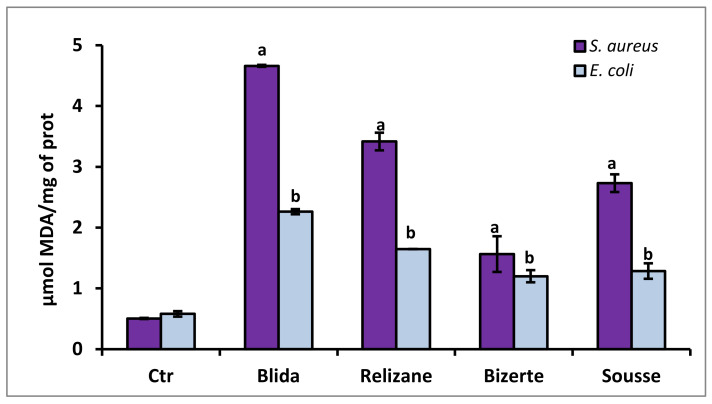
Determination of the production of malondialdehyde MDA (µmol MDA/mg prot) in *E. coli* and *S. aureus* in the presence of *M. azedarach* leaf extracts. CTR: control. MDA levels are expressed as mean ± SD. Comparisons of MDA rates between CTR and *E. coli* or *S. aureus* treated with leaf extracts from each studied region were performed using the unpaired two-tailed Student’s *t*-test. Significant differences among tested groups are indicated by different letters. ^a^ *p* < 0.001: *E. coli* values are significantly different from CTR; ^b^ *p* < 0.001: *S. aureus* values are significantly different from CTR.

The increase in lipid peroxidation marked by the production of malondialdehyde (MDA) is one of the side effects of the increased production of ROS. Therefore, we quantified the concentration of MDA in the treated bacteria (Figure 4).

An increase in MDA levels was observed compared with the control after the treatment of *S. aureus* and *E. coli* strains with various plant extracts. The extract from Blida showed the greatest increase in MDA levels; they were nine times higher than the control for the *S. aureus* strain and four times higher than the control for the *E. coli* strain.

### 3.7. Evaluation of Production of Superoxide Dismutase (SOD) and Catalase (CAT)

**Figure 5:**
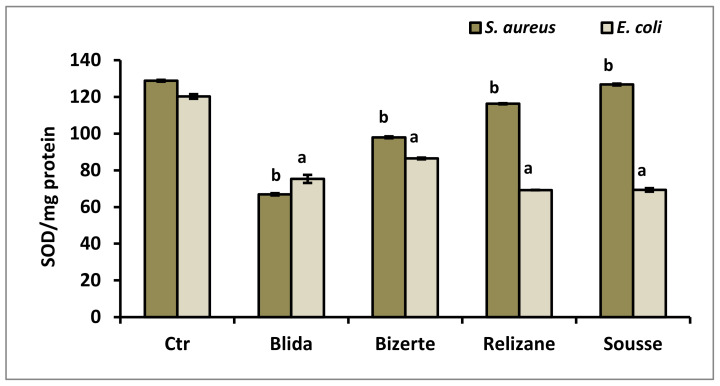
Determination of SOD activity (SOD/mg protein) in *E. coli* and *S. aureus* in the presence of *M. azedarach* leaf extracts. CTR: control. Data are reported as mean ± SD. SOD activity comparisons between CTR and *E. coli* or *S. aureus* treated with leaf extracts from each studied region were performed using the unpaired two-tailed Student’s *t*-test. Significant differences among the tested groups are indicated by different letters. ^a^ *p* < 0.001: *E. coli* values are significantly different from CTR; ^b^ *p* < 0.001: *S. aureus* values are significantly different from CTR.

Superoxide dismutase activity was assessed following the treatment of two bacterial strains, *S. aureus* and *E. coli*, with various *M. azedarach* extracts. This enzyme is important in the defense of the bacterial cell against oxidative stress. Figure 5 displays the outcomes of the test.

Our results revealed that the highest SOD activities were observed in the untreated bacteria, which served as controls for the *S. aureus* (128 SOD/mg protein) and *E. coli* (120 SOD/mg protein) strains. Treatment with the different *M. azedarach* extracts revealed a significant decrease in this antioxidant activity, with the extract from the Blida region showing the greatest decrease (66 SOD/mg protein) compared to the *S. aureus* control, while the extracts from the Tunisian regions of Bizerte and Sousse showed a considerable decrease in SOD levels (69 SOD/mg protein) compared to the *E. coli* control.

**Figure 6:**
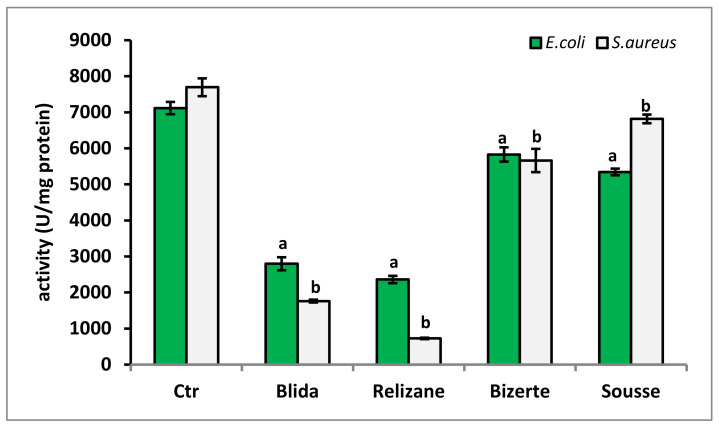
Determination of CAT activity (U/mg protein) in *E. coli* and *S. aureus* in the presence of *M. azedarach* leaf extracts. CTR: control. Data are reported as mean ± SD. CAT activity comparisons between CTR and *E. coli* or *S. aureus* treated with leaf extracts from each studied region were performed using the unpaired two-tailed Student’s *t*-test. Significant differences among the tested groups are indicated by different letters. ^a^ *p* < 0.001: *E. coli* values are significantly different from CTR; ^b^ *p* < 0.001: *S. aureus* values are significantly different from CTR.

For catalase activity, our results also showed that the highest rate of production of the CAT enzyme was recorded in untreated bacteria: 23,524 U/mg protein for the *E. coli* strain and 7694 U/mg protein for the *S. aureus* strain (Figure 6). After treatment of the bacterial strains with the different *M. azedarach* extracts, a significant decrease in CAT activity was observed for *E. coli*, with the extracts from the Blida and Relizane localities showing the greatest significant decreases of 2797 U/mg protein and 2360 U/mg protein, respectively, for the two extracts. These same plant extracts also showed the greatest reduction in CAT levels for the *S. aureus* strain, with a rate of 1763 U/mg of protein for the Blida region and 726 U/mg of protein for the Relizane locality.

## 4. Discussion

Extensive research using various analytical techniques has been carried out on several parts of *M. azedarach* and has led to the identification of different phenolic compounds and their derivatives, which is in line with our results. Despite the exploitation of plant-based bioactive compounds for various applications [31], the use of these molecules to combat pathogenic bacteria and boost the effectiveness of conventional antibiotics has long gone unnoticed [32]. In our study, in order to highlight the importance of these bioactive compounds, we analyzed the phytochemical composition of four methanol extracts of *M. azedarach* from different geographical origins (Algerians and Tunisians) and noted quantitative differences between their phenolic profiles.

Our results showed that methanolic leaf extracts from Algerian plants showed an abundance of gallic acid, chlorogenic acid, and caffeic acid. Quercetin and isorhamnetin were present in smaller quantities in the studied plants. Anterior investigations expanded our knowledge of the phenolic compounds in this plant; we used different analytical techniques to reveal the presence of several compounds. These included rutin and quercetin, which were identified by high-performance thin-layer chromatography (HPTLC); gallic acid, caffeic acid, and naringenin, detected by high-pressure liquid chromatography (HPLC) [33]; andkaempferol-3-O-robinobioside, kaempferol-3-O-rutinoside, and isoquercitrin, isolated from leaf methanol extracts of *M. azedarach* by column chromatography [34]. Other related compounds have also been identified in leaf extracts of this plant, such as chlorogenic conjugates, p-coumaric conjugates, gentisic conjugates, kaempferol conjugates, quercetin conjugates, chlorogenic acid [35], and quercetin-3-O-rutinoside [36]. These variations in chemical composition can be explained by the differences in the geographic origins of the samples, including the growing environment, due to factors such as soil, temperature, altitude, and precipitation [37]. The observed differences can also be attributed to the examined plant organs, and the extraction solvents which were utilized, and the analytical conditions used to perform this analysis [38]. All of these variables influence the chemical profiles of total phenolic compounds and, therefore, their biological activity [6].

Numerous studies have attributed the therapeutic efficacy of medicinal and aromatic plants to their bioactive phytocomponents, such as terpenoids, polyphenols, and alkaloids [39]. In this context, following the chemical composition analyses, we evaluated the antibacterial properties of *M. azedarach* extracts from the four selected regions. The results of antibacterial activity evaluated by the disc diffusion method have demonstrated the effectiveness of the extracts tested against Gram-positive bacterial strains with inhibition zones of a minimum diameter of 10 mm. The antibacterial activity of these extracts, expressed as minimum inhibitory concentration (MIC) and bactericidal concentration (BMC), was in correlation with the results of the obtained inhibition diameters. It was found that the extracts with the broadest inhibition spectra were also those with the lowest MIC values for the corresponding bacterial strains. In addition, the results showed that the methanol extracts of *M. azedarach* were less active against Gram-negative bacterial strains, with smaller inhibition diameters and higher MIC values. This is in agreement with previous reports showing that the methanol extracts exhibited higher antimicrobial activity against Gram-positive bacteria [40]. This activity of plant extracts is attributed to their secondary metabolites, and mainly to polyphenolic compounds [38].

Bacterial resistance to various antimicrobial agents is promoted by microbial biofilms. Therefore, the evaluation of the biofilm inhibition potentials of bioactive substances remains intriguing. The antibiofilm effects of the studied leaf extracts of *M. azedarach* revealed that the tested extracts had different effects on the growth and development of preformed biofilms in a dose-dependent manner. In fact, the strains treated with a concentration of MIC × 4 showed the highest rates of eradication of the biofilm, and the most important activity was obtained with the leaf extract from Relizane against the *S. aureus* and *E. coli* strains. More generally, we observed that samples from Algerian localities showed relatively higher eradication rates than other samples (from Tunisia). This difference could be attributed to the specific climatic conditions and environments of these regions, as it could be due to quantitative variation in the plant’s chemical compounds. Indeed, the culture medium could influence the biosynthesis of secondary metabolites, including the polyphenols responsible for this antibacterial effect [37]. Previously, the efficacy of plant methanol extracts against preformed biofilms of pathogenic bacteria has been reported [41,42]. In fact, plant phenolic compounds are responsible for the antibiofilm effect by influencing the mechanisms of bacterial regulation, including the quorum detection system [43]. For example, flavonoids, particularly quercetin, a compound present in the leaf extracts of *M. azedarach*, have been described to reduce and inhibit biofilm formation by decreasing the autoinducer-activated intracellular signaling responsible for intercellular communication [44].

Different pathways have been underlined regarding the phenolic compounds’ antibacterial action. Here, we assessed the impact of *M. azedarach* extracts on the alteration of the membrane integrity of the treated bacteria. As a result, the tested components (from all regions) induced disruption of the cell membrane in both Gram-positive and Gram-negative strains, leading to the release of cytoplasmic content. This action was revealed by an increase in the absorbance of the supernatant at 260 nm and 280 nm, revealing the release of nucleic acids and proteins from bacterial cultures [40]. As a result, bacterial exposure to *M. azedarach* extracts triggered the leakage of proteins and nucleic acids out of the bacterial cytoplasm, indicating a loss of membrane integrity and permeability [45]. The mechanisms of phenolic compounds’ antibacterial activity are diverse; they can interact with bacterial cell wall structures and can damage cytoplasmic membranes, such as catechins, which have been found to be able to penetrate and interact with lipid bicouches, causing membrane fusion and leakage of intra-membranous materials [43]. Additionally, polyphenols such as triterpenes, coumarins, quinones, and tannins can also affect membrane fluidity and inhibit the synthesis of nucleic acids, leading to their antibacterial efficacy against Gram+ and Gram− bacteria [46].

Indeed, the antibacterial activity can also be explained by the fact that phenolic acids such as gallic acid, caffeic acid, and chlorogenic acid, found in our various leaf extracts, are known to interact with membrane lipids by neutralizing their electrical potential as well as creating a complex with bacterial cell walls. On the other hand, these polyphenols can establish hydrogen bonds with proteins or enzymes of the cell wall, inhibit bacterial metabolism, or even sequester substances necessary for the growth of bacteria [47]. A previous study by Li et al. showed that exposure of *S. aureus* to chlorogenic acid results in cell membrane alterations [48]. This explains why the loss of integrity of the bacterial membrane following exposure to our leaf extracts would probably be due to the significant presence of these different compounds.

One of the mechanisms of action of phenolic compounds is the induction of oxidative stress in the bacterial cell as a result of enhancement of reactive oxygen species (ROS) production [49]. Our results revealed that methanolic extracts of *M. azedarach*, especially from Algerian localities, exhibited cytotoxic effects on *S. aureus* and *E. coli* through ROS generation compared to untreated strains. Furthermore, previous findings have reported the ability of catechin and isorhamnetin to penetrate bacterial cell membranes and induce oxidative stress through intracellular ROS production, resulting in their observed antibacterial potential [50]. ROS are considered intracellular apoptotic factors in treated bacteria because they induce the oxidation of macromolecules (proteins, lipids, DNA), leading to bacterial cell death [51]. Furthermore, lipid peroxidation is one of the side effects of high ROS in cells. In the present study, we quantified the levels of malondialdehyde (MDA), which has been extensively employed as a reliable biomarker for assessing lipid peroxidation of fatty acids [52]. Our results showed that extracts from Algerian regions caused a greater increase in MDA levels compared to Tunisian extracts. Likewise, the increase in lipid peroxidation registered in the treated strains was correlated with the production of ROS in these strains [16].

Cellular responses to oxidative stress can be measured by monitoring the activity of antioxidant enzymes such as catalase (CAT) and superoxide dismutase (SOD). In fact, the SOD enzyme catalyzes the conversion of highly reactive superoxides into oxygen and hydrogen peroxide. The peroxide is then lysed by the CAT enzyme in molecular oxygen and water [53,54]. Our findings revealed that *M. azedarach* leaf extracts decreased the antioxidant enzymes CAT and SOD in both the Gram-positive and Gram-negative bacteria which was tested. Other studies have shown a decrease in catalase activity in *S. aureus* when it is exposed to various plant chemicals, such as silibin and catechin, which is consistent with our findings [55]. Indeed, catalase produced by bacteria facilitates cell detoxification, which allows them to repair or escape oxidative damage caused by H_2_O_2_. The reduction in catalase activity caused by biologically active substances could lead to an increase in H_2_O_2_ levels and to oxidative stress-mediated toxicity in bacterial cells [51].

## 5. Conclusions

Based on the phytochemical characterization of *M. azedarach* leaf extracts from four localities, along with their antimicrobial activities, our study demonstrated that the evaluated methanolic extracts demonstrated potent antibacterial activities against Gram-positive and Gram-negative bacteria. Furthermore, the tested substances exerted substantial antibiofilm activities, influenced membrane integrity, and elevated ROS generation in pathogenic bacterial strains, resulting in lipid peroxidation and antioxidant enzyme activity suppression. Our results highlight the potentialities of *M. azedarach* extract and emphasize its valorization for the development of new anti-infectious agents.

## Figures and Tables

**Table 1:** Identification of bioactive compounds detected in methanolic extracts of *M. azedarach* leaves from different localities.

Peak	Rt (min)	Chemical Compounds	Bizerte (µg/100 mL)	Sousse (µg/100 mL)	Relizane (µg/100 mL)	Blida (µg/100 mL)
1	2.64	Gallic acid	6172.11 ± 34.48 ^a,b,c^	5121.29 ± 10.06 ^d,e^	6536.38 ± 20.98 ^f^	7601.04 ± 45.51
2	6.06	Chlorogenic acid	1257.62 ± 33.92	827.32 ± 16.99 ^d,e^	1527.56 ± 47.60	1529.57 ± 57.21
3	7.36	Caffeic acid	1298.99 ± 47.29 ^a,b,c^	1493.66 ± 50.78 ^d,e^	3761.98 ± 32.54 ^f^	3395.71 ± 64.66
4	16.64	Hyperoside	182.08 ± 1.67 ^a,b^	371.45 ± 1.06 ^d,e^	72.83 ± 1.05 ^f^	179.85 ± 1.50
5	17.70	Isoquercetin	429.44 ± 0.48 ^a,b,c^	1054.13 ± 0.60 ^d,e^	140.43 ± 1.55 ^f^	148.71 ± 1.02
6	31.53	Quercetin	Trace	22.22 ± 0.71 ^d,e^	11.85 ± 0.90 ^f^	5.38 ± 0.96
7	41.79	Isorhamnetin	7.41 ± 0.59 ^a,b,c^	10.55 ± 0.57 ^d,e^	19.61 ± 1.17 ^f^	16.22 ± 0.78

Values of phenolic compounds are represented as means ± SD of three measurements, and were statistically compared by one-way ANOVA followed by Tukey’s multiple comparisons tests. ^a^ *p* < 0.001: significantly different from Sousse; ^b^ *p* < 0.001: significantly different from Relizane; ^c^ *p* < 0.001: significantly different from Blida; ^d^ *p* < 0.001: significantly different from Relizane; ^e^ *p* < 0.001: significantly different from Blida; and ^f^ *p* < 0.001: significantly different from Blida.

**Table 2:** Inhibition zones of different *M. azedarach* extracts against pathogenic bacteria.

Origin	Concentration	*S. aureus*	*S. epidermidis*	*E. coli*	*P. aeruginosa*
Blida	150 (mg/mL)	13.5 ± 0.70 **	13 ± 0.04 ***	11.5 ± 0.7 **	12.5 ± 0.35
	300 (mg/mL)	16 ± 0.08	16.5 ± 0.70	13.75 ± 0.35	13.5 ± 0.70
Relizane	150 (mg/mL)	9.75 ± 1.06 *	14.25 ± 1.06 ***	13 ± 0.06 ***	9 ± 0.02 *
	300 (mg/mL)	12.5 ± 0.70	19 ± 0.03	16.7 ± 0.70	10.5 ± 0.70
Bizerte	150 (mg/mL)	12.25 ± 0.35 **	9.5 ± 0.70 **	12.75 ± 0.35 ***	8.5 ± 0.70 **
	300 (mg/mL)	15.5 ± 0.70	12.8 ± 0.28	14.5 ± 0.70	11 ± 0.02
Sousse	150 (mg/mL)	9.5 ± 0.70 *	10.5 ± 0.70 **	10 ± 0.08 **	8 ± 0.05 **
	300 (mg/mL)	11.75 ± 0.35	13 ± 0.07	12 ± 0.09	9.5 ± 0.70
GEN	(10 µg)	24	24	25	22

Comparison between increasing concentrations of leaf extracts from each studied region was performed using the unpaired two-tailed Student’s *t*-test. * *p* < 0.05, ** *p* < 0.01, and *** *p* < 0.001 indicate significant differences from the 300 mg/mL concentration. GEN: Gentamicin antibiotic.

**Table 3:** Results of the minimal inhibitory and bactericidal concentrations of *M. azedarach* from different provenances.

Origin	MIC/MBC (mg/mL)	Bacterial Strains			
		*S. aureus*	*S. epidermidis*	*E. coli*	*P. aeruginosa*
Blida	MIC	31.25	31.25	31.25	125
	MBC	>250	>250	>250	>250
Relizane	MIC	62.5	31.25	31.25	125
	MBC	>250	>250	>250	>250
Bizerte	MIC	62.5	62.5	31.25	125
	MBC	>250	>250	>250	>250
Sousse	MIC	125	62.5	62.5	125
	MBC	>250	>250	>250	>250

MIC: minimum inhibitory concentration; CMB: minimum bactericidal concentration.

**Table 4:** Percentages of biofilm eradication after treatment with various concentrations of *M. azedarach* leaf extracts.

Origin	Concentration	Biofilm Eradication (%)			
		*S. aureus*	*S. epidermidis*	*E. coli*	*P. aeruginosa*
Blida	MIC	21.46 ± 2 ^a,b^	19.01 ± 2.3 ^a,b^	17.73 ± 0.7 ^a,b^	16.22 ± 1.4 ^a,b^
	MIC × 2	46.23± 1.4 ^c^	42.05 ± 1.5 ^c^	28.47 ± 0.5 ^c^	34.15 ± 2.7 ^c^
	MIC × 4	65.52 ± 1.9	53.49 ± 3	47.55 ± 1.5	44.65 ± 2
Relizane	MIC	15.47 ± 2.7 ^a,b^	21.12 ± 3.2 ^a,b^	32.66 ± 1.2 ^a,b^	11.79 ± 4.9 ^a,b^
	MIC × 2	50.47 ± 2.3 ^c^	41.54 ± 3.2 ^c^	40.73 ± 1.3 ^c^	51.4 ± 4.3 ^c^
	MIC × 4	72.17 ± 2.2	53.72 ± 0.4	60.26 ± 4.7	55.56 ± 0.2
Bizerte	MIC	17.15 ± 0.8 ^a,b^	22.02 ± 2.4 ^a,b^	8.37 ± 1.3 ^a,b^	6.29 ± 2.7 ^a,b^
	MIC × 2	39.05 ± 0.6 ^c^	28.11 ± 2.0 ^c^	22.97 ± 1.8 ^c^	23.28 ± 2.4 ^c^
	MIC × 4	47.22 ± 0.6	36.61 ± 0.5	33.37 ± 1.7	50.51 ± 2.1
Sousse	MIC	16.13 ± 1.1 ^a,b^	17.4 ± 7.6 ^a,b^	23.25 ± 2.1 ^a,b^	26.65 ± 2.5 ^a,b^
	MIC × 2	34.65 ± 1.1 ^c^	26.29 ± 7.7 ^c^	35.08 ± 0.2 ^c^	46.68 ± 2.7 ^c^
	MIC × 4	49.38 ± 1	57.4 ± 1.9	47.56 ± 1.5	59.95 ± 0.6

Biofilm eradication values are represented as mean percentages ± SDs of three independent measurements, and were statistically compared by one-way ANOVA followed by Tukey’s multiple comparisons tests. ^a^ *p* < 0.001: significantly different from MIC × 2; ^b^ *p* < 0.001: significantly different from MIC × 4; ^c^ *p* < 0.001: significantly different from MIC × 4.

## Data Availability

Not applicable.
